# Organotypic culture as a research and preclinical model to study uterine leiomyomas

**DOI:** 10.1038/s41598-020-62158-w

**Published:** 2020-03-23

**Authors:** Ana Salas, Judith López, Ricardo Reyes, Carmen Évora, Francisco Montes de Oca, Delia Báez, Araceli Delgado, Teresa A. Almeida

**Affiliations:** 1 Departamento de Bioquímica, Microbiología, Biología Celular y Genética, Universidad de La Laguna. Facultad de Ciencias. Sección de Biología. Avda. Astrofísico Fco. Sánchez s/n, 38200 San Cristóbal de La Laguna, Tenerife Spain; 2 Instituto de Enfermedades Tropicales y Salud Pública de Canarias (IUETSPC). Avda. Astrofísico Fco. Sánchez s/n, 38200 San Cristóbal de La Laguna, Tenerife Spain; 3 Departamento de Ingeniería Química y Tecnología Farmacéutica, Universidad de la Laguna, Tenerife, Spain. Avda. Astrofísico Fco. Sánchez s/n, 38200 San Cristóbal de La Laguna, Tenerife Spain; 4 Instituto de Tecnologías Biomédicas (ITB). C/Sta. María Soledad, s/n. Facultad de Ciencias. Sección de Medicina, 38200 San Cristóbal de La Laguna, Tenerife Spain; 5 Hospital Quironsalud, C/Poeta Rodríguez Herrera 1, Santa Cruz de Tenerife, Tenerife 38006 Spain; 6 Departamento de Obstetricia y Ginecología, Facultad de Ciencias de La Salud, Universidad de La Laguna, Campus de Ofra s/n, San Cristobal de La Laguna, Tenerife Spain

**Keywords:** Cancer models, Gene expression

## Abstract

Organotypic cultures of tissue slices have been successfully established in lung, prostate, colon, gastric and breast cancer among other malignancies, but until now an *ex vivo* model based on tissue slices has not been established for uterine leiomyoma. In the present study, we describe a method for culturing tumour slides onto an alginate scaffold. Morphological integrity of tissue slices was maintained for up to 7 days of culture, with cells expressing desmin, estrogen and progesterone receptors. Driver mutations were present in the *ex vivo* slices at all-time points analyzed. Cultivated tumour slices responded to ovarian hormones stimulation upregulating the expression of genes involved in leiomyoma pathogenesis. This tissue model preserves extracellular matrix, cellular diversity and genetic background simulating more *in-vivo-like* situations. As a novelty, this platform allows encapsulation of microspheres containing drugs that can be tested on the *ex vivo* tumour slices. After optimizing drug release rates, microspheres would then be directly tested in animal models through local injection.

## Introduction

Uterine leiomyoma (UL), also called fibroid or myoma, is the most commonly diagnosed tumour of the female genital tract with an incidence of 40% at the age of 35 and nearly 70–80% around the age of 50^1,2^. Severe symptoms develop in 15–30% of patients being irregular, prolonged or heavy vaginal bleeding the most common manifestations frequently associated with moderate to severe anemia^1^. In addition, UL may interfere with embryo implantation, complications during pregnancy and childbirth, decreasing reproductive success^3^. Fibroids are the leading cause of hysterectomy with an estimated US healthcare cost of ~$21 billion annually^4^.

Although the etiology remains elusive, some progress has been made about the molecular mechanisms involved in tumour initiation and growth including (1) steroid hormone–dependency^2^, (2) excessive accumulation of extracellular matrix (ECM), a reservoir for growth factors, cytokines, chemokines, angiogenic and inflammatory response mediators and proteases that contribute to cell growth, differentiation and ECM turnover^5,6^ and (3) alterations in driver genes such as *HMGA2* (high mobility group AT-hook 2) and *MED12* (mediator complex subunit 12) occurring in most of the tumours^7,8^. Different cell populations are found in leiomyomas, including smooth muscle (SMC), fibroblast and stem cells^9–12^, all of them embedded in the abundant ECM. Communication between these cells seems to be critical for tumour proliferation and survival^9–11^. Thus, it has been proposed that leiomyoma stem-progenitor cells devoid of sex hormonal receptors, respond to paracrine signals sent by surrounding tumour cells upon ovarian steroid stimulation, inducing self-renewal and tumour maintenance and growth^13^. Furthermore, UL derived fibroblast stimulate the proliferation of leiomyoma cells and collagen type I production^6,11,14^.

Most studies published to date have used traditional 2D culture models of primary or immortalized cells from SMCs, to understand the growth and behavior of UL and to look at factors that may affect fibroid growth^5,13^. However, this model neither capture three dimensional tumour architecture nor many of the important signalling dynamics of the interactions among all cells present in the tumour and between these and the microenvironment. In addition, rapid disappearance of cells carrying *MED12* mutation and decrease of estrogen and progesterone receptors expression as the days of culture progress have been observed, challenging the usefulness of 2D culture model^15–18^.

Organotypic cultures consist of sectioned tumour tissue into thin slices, mounted onto porous membranes for mechanical support and incubated in a controlled condition^19^. They retain histological and three-dimensional structure (3D), with inter- and extracellular interactions, cell matrix components, and intact metabolic capacity. This approach has been successfully used to gain insights into tumour biology and as a preclinical model for drug discoveries in many different cancers^19,20^.

Our present study aimed to develop a well-characterized 3D organotypic culture system using precision-cut slices from human uterine leiomyoma placed onto an alginate scaffold. In order to determine whether the freshly prepared explants are capable of capturing and then maintaining essential features of the original tumour, tissue slices were harvested at different time points and compared to the original tumour using histology and immunohistochemistry (IHQ). In addition, tumour slices were stimulated with ovarian steroids and selected transcripts and proteins were quantified by real time PCR (qPCR) and western blot, respectively.

## Results

### Alginate polymer constitutes a suitable scaffold for tissue culture explant

Tissue slices were cultured on 1 mm thickness alginate scaffold which allow preservation of 3D tissue structure in culture and position the tissue slice at the air/liquid interface enabling efficient oxygenation (Supplementary Figure S1). The high porosity of the alginate sponge (average 84%) with interconnected pore network^21^ enables the solutes diffusion through the scaffold allowing adequate supply of nutrients and oxygen to the cells. In addition, microsphere-incorporating scaffolds provide appropriated mechanical properties for *in vitro* culture. In our system, we obtained microspheres diameter mean volume of 59.31 µm (10% < 26.75 µm and 90% < 84.23 µm), with capacity for high drug loading yield and a tunable sustained release^22^, which would be ready for fast testing in animal models after the *in vitro* assay^23,24^.

### Morphology is preserved in organotypic tissue cultures

**Figure 1:**
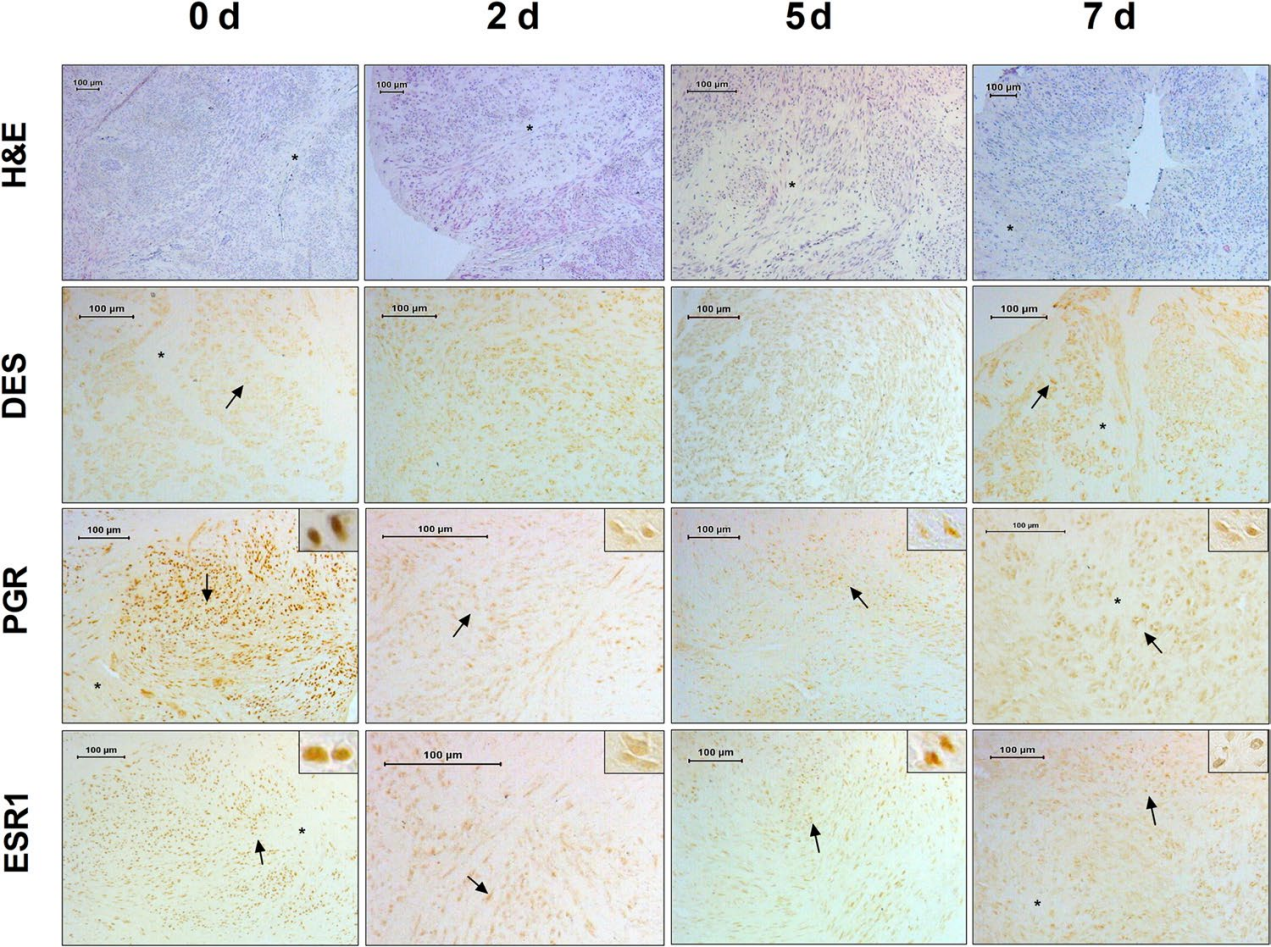
Histology and Immunohistochemistry analysis of UL and derived organotypic tissue cultures. Semi-panoramic images of a representative tumour at baseline (0 d) and thereafter at 2, 5 and 7 days of culture. H&E staining showed maintenance of tissue morphology after 7 days of culture. Immunoreaction for desmin (DES) confirm the smooth muscle nature of most of the cells present in the tissue. Abundant immunoreaction for progesterone (PGR) and estrogen receptor (ESR1) was observed during 7 days of culture. The inserts (top right) show immunoreaction in the nucleus of the smooth muscle cells. Asterisks indicate disorganized ECM. Arrows indicate specific immunoreaction. Scale bar 100 µm.

Slices from tumours were maintained in culture for up to 3 weeks and tumour cells morphology was assessed by H&E staining. Morphological integrity of tissues, defined as preservation of general architecture was confirmed in the tumour samples up to 7–10 days of culture. Typical UL histology was observed in the original tumour and derived tissue culture slices, with the presence of areas of smooth muscle fascicles arranged in different orientations surrounded by variable degrees of ECM (Fig. 1). After 1 week of culture, a progressive decrease in cellularity with a concomitant increase in ECM was observed.

Abundant immunoreactivity (-ir) for the muscle marker desmin was found in T0 and the cultured tumour slices (Fig. 1). Similarly, we detected estrogen and progesterone receptors at all-time points analyzed, with a clear signal observed in the nuclei of the smooth muscle cells (Fig. 1). We analyzed the number of immunoreactive cells across different time points (T0, T2, T7, T10) in 3 tumours that preserved tissue morphology for up to 10 days as assessed by H&E staining (Supplementary Figure S2). In one of the tumours DES-ir, ESR1-ir and PGR-ir decreased significantly after 10 days of culture. Significant decreased in ESR1 and PGR signal was also detected after 10 days of culture in tumour L6 and L9, respectively (Supplementary Figure S2). According to these results, ex vivo slices of fibroids could be kept in culture for up to 7 days.

### Driver mutations are maintained in organotypic tissue cultures

**Figure 2:**
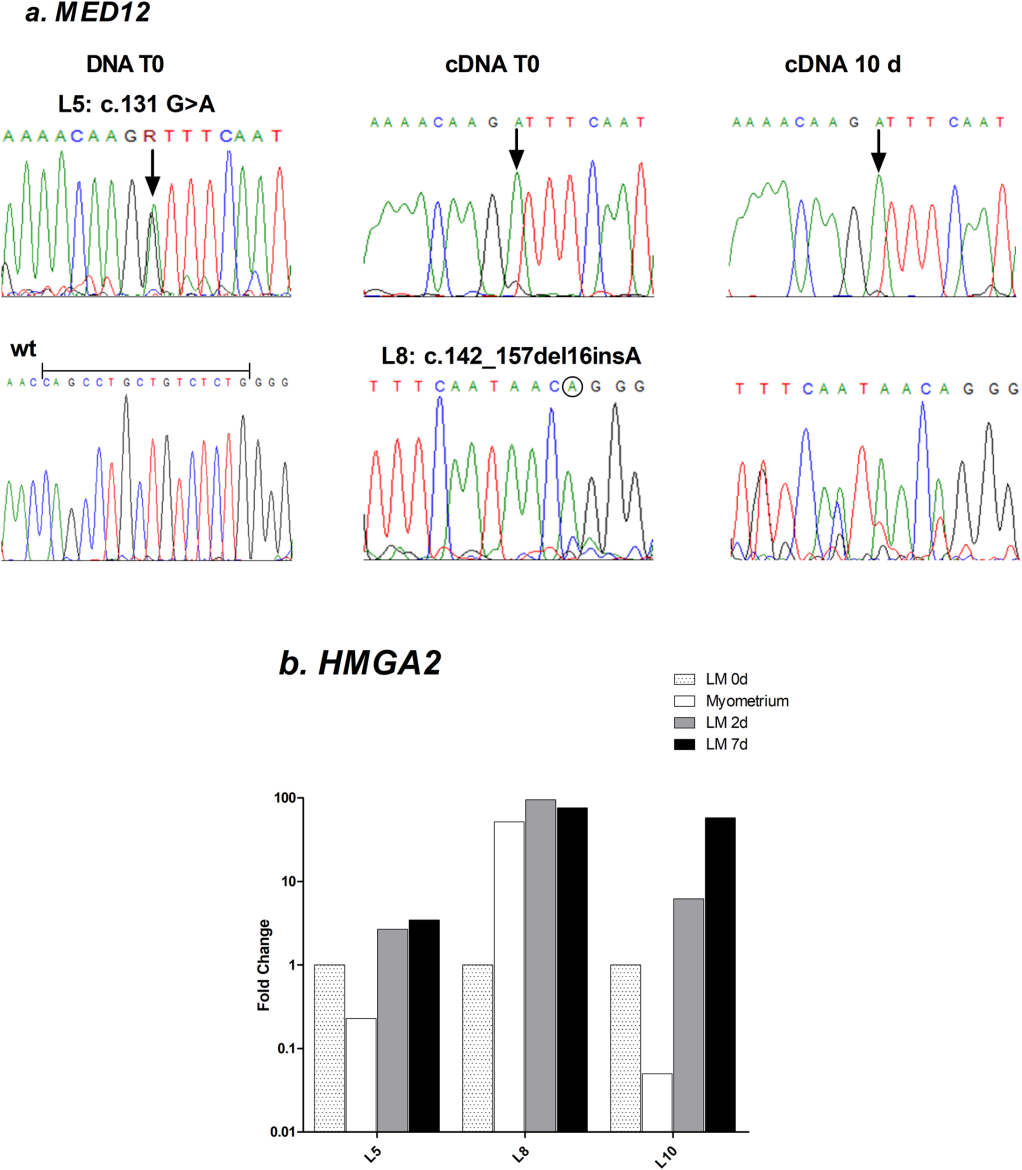
Driver mutations are maintained in organotypic tissue cultures. (**a**) sequence electropherograms illustrating different types of somatic mutations in *MED12*. Top line: DNA sequencing showed heterozygous mutation corresponding to a single base substitution in tumour L5 (arrow). cDNA sequencing showed predominant expression of mutant allele in original tumour and at 10 days of culture. Bottom line: Wild type sequence (wt) of *MED12* exon 2. cDNA sequencing showed an A insertion (circled) followed by a 16 pb in-frame deletion (underlined in wt sequence) in original L8 tumour and after 10 days of culture. (**b**) Relative quantification of *HMGA2* mRNA in 3 UL (L5, L8 and L10) at 2 and 10 days of culture compared to tumour at baseline (LM 0d). Expression of matched myometrium compared to LM 0d is also shown. Fold-changes are shown on Log10 scale.

We sequenced the exon 2 hot-spot region of *MED12* in the 11 UL analyzed and found mutation in 8 tumours (Supplementary Table S1). DNA sequencing showed that mutations were heterozygous consisting in seven single base substitutions and one in-frame deletion. cDNA sequencing demonstrated that the mutant allele was predominantly expressed in T0 and after 10 days of culture in both point mutation and deletion (Fig. 2a).

Of the tumours employed for long term culture, relative quantification of *HMGA2* mRNA in original tumour samples demonstrated upregulation in two tumours (L5, L10) compared to matched myometrium, while tumour L8 showed increased levels in myometrium (Fig. 2b). These 3 tumours increased considerably *HMGA2* mRNA levels, 3.5-, 76- and 57-fold after 7–10 days of culture compared to T0 (Fig. 2b). The remaining 3 tumours showed absent or weak expression of *HMGA2* mRNA in T0 and during culture.

### Cultivated tumour slices responded to ovarian hormones stimulation across time

**Figure 3:**
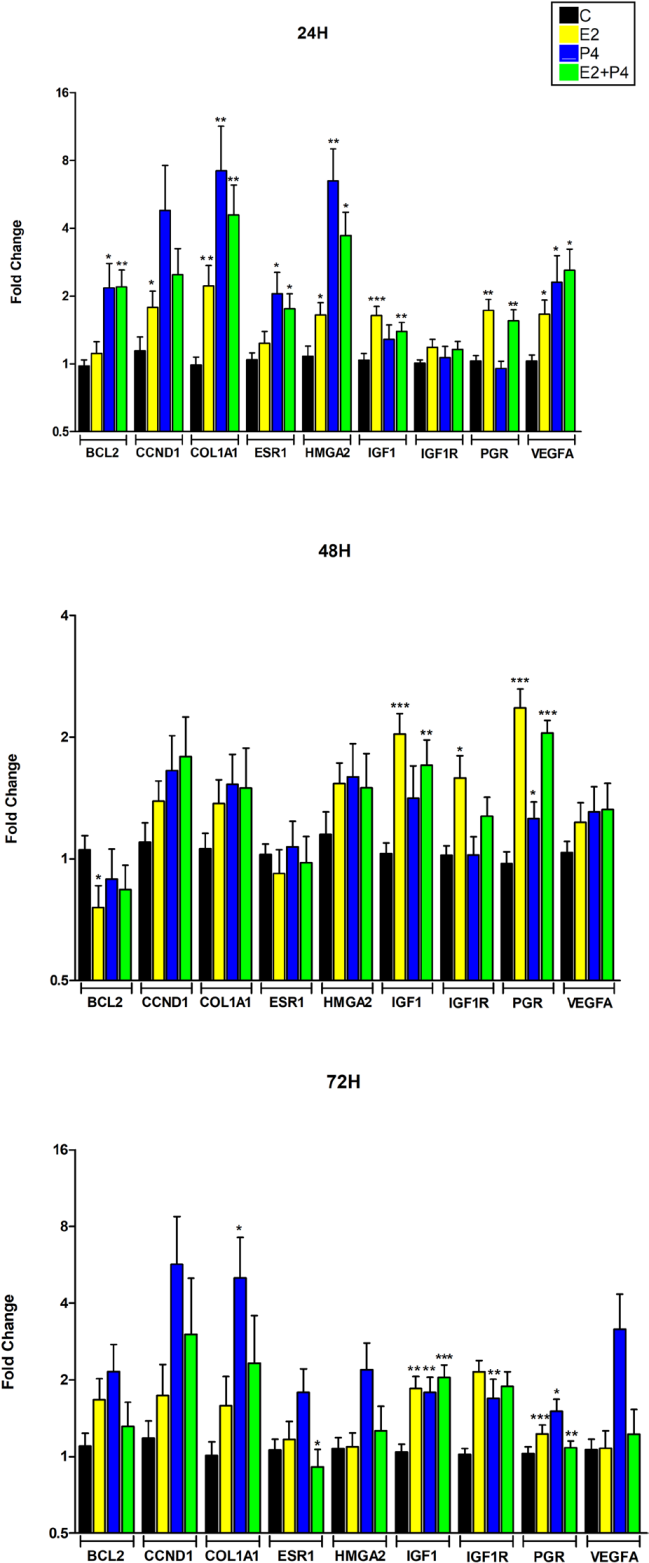
Differential expression of hormone responsive genes during slice culture measured by qPCR. Gene expression profile of 5 different tumours after stimulation with 17-β-estradiol (E2), progesterone (P4) or both (E2 + P4) during 24, 48 and 72 hours. Bars show the mean fold change, and error bars indicate standard error of the mean (SEM). Fold-changes are shown on Log 2 scale. *BCL2* (apoptosis regulator) *CCND1* (cyclin D1), *COL1A1* (collagen type I alpha 1 chain), *ESR1* (estrogen receptor 1), *HMGA2* (high mobility group AT-hook 2), *IGF1*(insulin like growth factor 1), *IGF1R* (insulin like growth factor 1 receptor), *PGR* (progesterone receptor, both isoforms), *VEGFA* (vascular endothelial growth factor A). *p < 0.05, **p < 0.01, *** p < 0.001.

In order to determine whether tumour slices responded to ovarian steroids, *ex vivo* slices from 5 tumours were culture in presence of estradiol, progesterone or a combination of both during 24, 48 and 72 hours and gene-expression profiling of 8 selected hormone responsive genes previously shown to be important for UL pathogenesis were analyzed by qPCR (Fig. 3, Table 1). We observed that tumour slices responded significantly to hormone stimulation increasing expression of selected genes at 24 h, 48 h and 72 h. (Fig. 3). In general, steroids-induced gene transcription was more evident at 24 and 72 hours compared to 48 hours. Interestingly, marked upregulation of *HMGA2* was observed with E2, P4 or combinations of both in the tumours analyzed at 24 hours. According to driver alterations, *MED12* was mutated in 4 tumours (*MED12*^*mut*^) while *HMGA2* upregulation was detected in tumour L23 (Supplementary Table S1). When gene expression data from both tumour types were compared, particular expression patterns were observed (Table 1). Thus, E2 stimulated the expression of few genes in MED12^mut^, particularly *IGF1* and *PGR* that showed high expression at all-time points analyzed. However, higher number of genes and stronger response to E2 was observed in L23 slices (Table 1). Progesterone upregulated the expression of many genes in both groups at 24 h and 72 h, but the combined effect of both hormones was more evident in L23 slices compared to MED12^mut^ group.

**Table 1:** mRNA Fold-change for 8 genes regulated by steroid hormones and the driver gene *HMGA2* in cultured slices. For each leiomyoma, normalized relative expression of the target gene was compared to its control slice. Average of 3 biological replicates for L23 is shown. MED12^mut^ include the mean fold of the 12 biological replicates from the 4 fibroids with MED12 mutation. Genes differentially expressed  ≥2 fold of the 4 tumors samples are indicated in bold. *p < 0.05, **p < 0.01, ***p < 0.001.

		SAMPLES	BCL2	CCND1	COL1A1	ESR1	HMGA2	IGF1	IGF1R	PGR	VEGFA
**24 H**	**E2**	**MED12**^**mut**^	0,98	1,33	1,5*	1,09	1,52	1,51***	1,14	1,79**	1,35
		**L23**	1,63	**3,59**	**5,06**	1,81	**2,14**	**2,11**	1,33	1,47	**2,90**
	**P4**	**MED12**^**mut**^	2,32*	**5,25**	7,93*	**2,09**	5,0*	1,15	0,98	0,95	**2,17**
		**L23**	1,60	**3,10**	**4,58**	1,88	**12,33**	1,77	1,39	0,99	**2,75**
	**E2** + **P4**	**MED12**^**mut**^	1,61*	1,41	3,45*	1,32	2,66^*^	1,39*	1,24*	1,26*	1,89
		**L23**	**4,52**	**6,44**	**9,09**	**3,50**	**9,99**	1,40	0,79	**2,70**	**5,45**
**48 H**	**E2**	**MED12**^**mut**^	0,72*	1,48*	1,54	1,01	1,53	2,18***	1,74**	**2,66**	1,35*
		**L23**	0,89	1,04	0,76	0,59	1,54	1,49	0,95	1,17	0,78
	**P4**	**MED12**^**mut**^	0,88*	1,78	1,69	1,13	1,60	1,30	1,12	1,18	1,39
		**L23**	0,95	0,89	0,57	0,72	1,57	1,88	0,66	1,57	0,79
	**E2** + **P4**	**MED12**^**mut**^	0,88	**2,04**	1,69	1,05	1,62	1,59**	1,28	**2,09**	1,42
		**L23**	0,58	0,33	0,32	0,56	0,79	**2,14**	1,26	1,88	0,76
**72 H**	**E2**	**MED12**^**mut**^	1,36	1,27	1,21	0,94	1,10	1,79*	1,19	2,28***	1,02
		**L23**	**2,91**	**4,56**	**3,82**	**2,09**	1,05	**2,21**	1,37	1,62	1,45
	**P4**	**MED12**^**mut**^	1,74	**4,23**	**4,00**	1,25	1,29	1,48*	1,47*	1,84*	**2,39**
		**L23**	**3,69**	**14,42**	**11,07**	**3,72**	**5,51**	**2,86**	1,63	1,10	**6,25**
	**E2** + **P4**	**MED12**^**mut**^	0,99	0,86	1,01	0,77**	0,97	1,80**	1,06	1,97***	0,95
		**L23**	**3,23**	**15,94**	**10,23**	1,73	**3,04**	**3,02**	1,16	1,52	**2,84**

**Figure 4:**
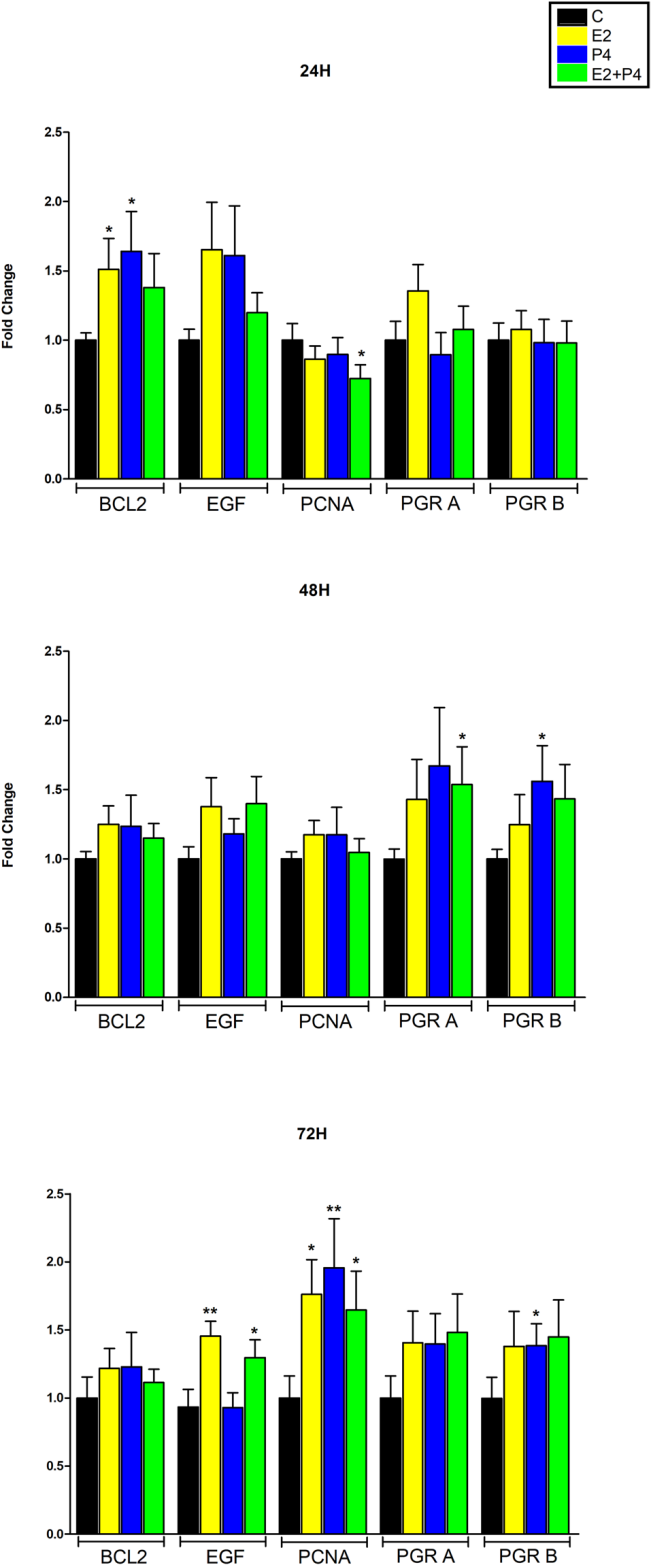
Regulation of protein expression during slice culture upon hormonal stimulation. Histogram showing densitometric analysis of protein bands from western blotting in ovarian steroids treated and control groups at 24, 48 and 72 hours. Proteins analyzed included proliferating cell nuclear antigen (PCNA), BCL2 apoptosis regulator, epidermal grow factor (EGF), progesterone receptor isoform A (PGRA) and progesterone receptor isoform B (PGRB). *p < 0.05, **p < 0.01. Each bar represents the mean fold change and error bars indicate standard error of the mean (SEM).

At protein level, we analyzed PCNA and BCL2 as protein markers for tumour proliferation and anti-apoptosis, respectively. Although at 24 h a slightly inhibitory effect on PCNA expression was observed after hormones addition, particularly with E2 + P4 (p = 0.02), a significant upregulation was observed after 72 hours with either combination (Fig. 4). At transcription level, we observed that Cyclin D1 (CCND1) whose activity is required for cell cycle G1/S transition, was significantly upregulated by E2 (p = 0.04) and close to significance by P4 (p = 0.06) and E2 + P4 (0.06) at 24 h and its transcript levels seem to remain upregulated at 48 h and 72 h (Fig. 3). E2 (p = 0.03) and P4 (p = 0.02) increased BCL2 protein expression after 24 hours of stimulus, with the same trend observed when both hormones were added (Fig. 4). This expression pattern was similar to that observed for mRNA, with significant upregulation observed after P4 (p = 0.02) and E2 + P4 (p = 0.0039) addition at 24 h.

Epidermal growth factor (EGF) has been demonstrated to play a crucial role as a local growth factor in regulating leiomyoma growth^25^. EGF showed a tendency to increase expression with all the ovarian steroids combination at 24 h, reaching significance at 72 h with E2 (p = 0.046) and E2 + P4 (p = 0.034).

Previous studies have demonstrated that estradiol induces expression of progesterone receptors (PGR) in leiomyoma cells^26^. At mRNA level, we found that E2 or E2 + P4 markedly induced the expression of PGR at all-time points analyzed (Fig. 3). From 48 h to 72 h, PGR mRNA levels were also upregulated after P4 stimulus. At protein level, single or combination of both hormones increased expression of both isoforms after 48 h and 72 h (Fig. 4).

It is worth noting the strong stimulatory effect of sex steroids on cultured slices in genes clearly involved in fibroids pathogenesis, such as *VEGFA*, *COL1A1* and *IGF*, the latter one being induced by E2 or E2 + P4 from 24 to 72 hours (Fig. 3).

## Discussion

We describe for the first time an optimized *ex vivo* organotypic culture of uterine leiomyoma. This approach has been successfully used in different cancers with tissue morphology and viability preservation for up to 5–7 days^19,27,28^. Here we show that culture of thin tissue explants from UL tumours maintain cell morphology for up to 7 days, and then a progressive decrease in cellularity, hormone receptors and increase in ECM was observed. In addition, slices cultured during 5 days responded to ovarian hormone stimulation regulating the expression of several genes at mRNA and protein level.

Since UL are firm, compact and very fibrotic tumours, it may be difficult to obtain thin sections from them consistently. In our experience, best results were achieved by cutting manually pieces of ~ 0.5-cm-wide × 0.5-cm-height before mounting them on the sectioning pedestal. In addition, a carbon steel blade with an angle of 26° was crucial to obtain complete slices and prevent the moving blade to dislodge the entire tissue piece.

Although tissue slices are usually placed on synthetic membrane inserts in 6-well plates commercially available, microspheres containing drugs can be integrated into scaffolds composed of organic materials, such as alginate, chitosan, collagen, PLGA, etc. After assessing the effect on *ex vivo* tumour slices and optimizing drug release rates, microspheres would then be tested in animal models through local injection.

Previous reports have detected rapid loss of *MED12* mutated cells upon *in vitro* growth of UL-derived cell^17,18^. Two possible explanations seem plausible, either absence of soluble environmental factors not present in culture media and essential for tumour growth, or loss of extracellular matrix and hence all the interactions among ECM and the tumoural cells^18^. However, in the organotypic culture we observed that both, point mutation and in-frame deletion alleles of *MED12*, preferentially expressed in original tumour, are maintained in derived cultures slices. Since we have used standard media previously used in 2D cultures, our results support the idea that ECM is fundamental for *in vitro* survival and growth of tumour cells carrying *MED12* mutations. In addition, organotypic culture may preserve the rate of fibroblasts to mutant smooth muscle cells, bypassing the weak adherence of SMC to plastic surface and hence avoiding its progressive loss during culture^14,18^.

We have observed that *HMGA2* mRNA progressively increased over time in cultured slices suggesting that the organotypic system enhance *HMGA2* driver alteration. In addition, we have seen for the first time that ovarian steroids increased *HMGA2* mRNA levels in the 5 UL tumours analyzed. High Mobility Group A (HMGA) genes encode DNA-binding non-histone proteins that control cell growth by indirect regulation of the DNA transcription process. The expression of HMGA2 is widely restricted to the embryonic stage and decreases to undetectable levels in adult tissues. HMGA2 expression in normal myometrial cells induces its transformation toward putative tumour-initiating leiomyoma cells^29^. In addition, disturbed expression in adult tissues has an important role in the growth of several mesenchymal tumours^30,31^. Based on our finding, we propose a new mechanism for leiomyogenesis where ovarian steroids induce, directly or indirectly, the expression of HMGA2 promoting LM cell proliferation and growth.

At the transcriptional level, tumours with *MED12* mutation barely respond to E2 stimulation (Table 1, Fig. 3) contrary to what was observed in slices from tumour without MED12 mutations. Since MED12 is a component of the mediator complex involved in transcription regulation, mutations in this gene may impair transcription response following ovarian steroids stimulation. In fact, it has been shown that *MED12* bind to *ESR1* gene promoter and together with cohesin promotes the expression of ERα^32^. *MED12* silencing reduced PolII occupancy at the *ESR1* promoter and *ESR1* mRNA expression. In our dataset, we observed that estrogen increase *ESR1* expression at 24 h and 72 h in L23 but not in MED12^mut^ (Table 1). Interestingly, after 72 hours of E2 + P4 stimulus, a significant decrease of *ESR1* mRNA expression was observed in MED12^mut^ (Table 1). These data strongly support that *MED12* plays a major role in *ESR1* expression and that mutations in this gene may impair estrogen-regulated transcription. In the hormonal study, we have only one tumour without *MED12* mutation, which is in agreement with the high frequency of alterations observed for this gene in UL (50–80%). Therefore, further studies are needed before any conclusion can be drawn.

Integrative genome-scale studies of UL harboring different driver genetic alterations, including those in *MED12* and *HMGA2* contributing to 80–90% of leiomyomas, have uncovered UL subtypes gene expression signatures^8^. In this study we have observed variable response to hormones stimulation in tumours with two different driver alterations, supporting that UL organotypic cultures maintain molecular mechanisms to respond differentially according to its genetic background. This heterogeneity in tumour response to hormones stimulation may reflect the remarkable variable response observed in patients, with UL showing increase, decrease or not change in size to progestin stimulation^33^.

In conclusion, organotypic cultures of UL preserve particular local conditions, such as EMC, cellular diversity, genetic background and autocrine/paracrine signals constituting a more biologically representative system to study molecular mechanism of tumour growth. In addition, alginate scaffold containing microspheres with encapsulated drug may constitute a personalized preclinical model to investigate tumour responses to drugs for future localized therapy. Indeed, cytotoxic responses to targeted therapies as well as classic chemotherapeutic agents have been predicted in organotypic tissue slices^19,27,28,34–37^.

## Methods

### Ethical statement

This study was approved by the Institutional Review Board of the Committee for Clinical Research Ethics of the University Hospital Complex from Canary island (CHUNSC_2018_63). Informed consent was obtained from the patients before the collection of any samples. All experiments handling human tissues were performed in accordance with Tenets of the Declaration of Helsinki.

### Tissue sampling

Nine female patients aged 41–58 years, admitted to Hospital Quironsalud between 2018 and 2019 were enrolled in this study. Tumours analyzed included 7 intramurals, 1 submucous and 3 subserous tumours, up to 4–12 cm in size, as well as the matched myometrial tissue. Myometrial samples were taken as far away as possible from leiomyomata. All patients were Caucasian and underwent hysterectomy for menorrhagia without any previous hormonal treatment for at least 3 months (Supplementary Table S1). The histopathological analysis using standard H&E staining and performed by a pathologist indicated tumours with benign histology with no sign of malignancy, nuclear atypia, mitotic figures or necrosis. After surgery, samples were immediately submerged in sterile Hank’s balanced salt solution (HBSS) supplemented with 0.25 µg/mL amphotericin B, 100 U/mL penicillin and 100 µg/mL streptomycin (Sigma-Aldrich Co., St. Louis, MO, USA). Tissue pieces were then transported to the laboratory and processed under sterile conditions. Samples were processed within 4 hours after surgery.

### Fresh tissue sectioning

Automated slicing was performed using a vibratome (Vibroslice NVSLM1, World Precision Instruments) after immobilizing the samples using cyanoacrylate glue. Tissue block was cut submerged in cold and sterile HBSS supplemented with antibiotics and antimycotic in a laminar flow hood. Tissue slices of 500 μm were obtained with the following instrument parameters: 1.0 mm of vibration amplitude (fixed), 0.3 to 0.5 mm/sec of slicing speed, 2000 rpm of vibration blade speed and 26° of blade angle.

### Scaffold preparation and characterization

Scaffolds consisting in a mixture of alginate and poly-lactic acid (PLA) blank microspheres were prepared under aseptic conditions. The microspheres were elaborated by a simple emulsion (o/w) and solvent evaporation method as previously described^22^. Briefly, a solution of 200 mg PLA (Resomer® R-203S, Evonik, Darmstadt, Germany) in methylene chloride (0,5 mL, DCM) was emulsified with 4 mL of 1% poly-vinyl alcohol aqueous solution (PVA, Mw = 30–70 kDa, Sigma-Aldrich, St. Louis, MO, USA) using vortex for 1 min (position 10, Genie^®^ 2, Scientific Industries, Inc, NY, USA), then poured into 100 mL of 0.16% PVA and left under magnetic stirring during 1 h for solvent evaporation. Afterwards, microspheres were collected by filtration (Supor^®^-450 filters, pore size 0.45 µm, Pall Corporation, Ann Arbor, MI, USA) and freeze-dried.

For scaffold preparation, microspheres (200 mg) were dispersed in 20 mL aqueous solution of 2% alginate (Pronova® UP MVG, NovaMatrix, Sandvika, Norway) in a petri dish (100 mm diameter) and freeze-dried. Then, alginate was cross-linked by adding CaCl_2_ 1% (20 mL) for 20 min, washed twice with water and freeze-dried again. Finally, individual scaffolds were obtained using a 6 mm diameter punch.

Microspheres size distribution was determined by laser diffractometry (Mastersizer 2000, Malvern Instruments, Malvern, UK). Porosity of the scaffolds was calculated as previously^23^ and scaffold thickness was measured from photographs obtained by stereo-microscopy (Leica M205C, Leica LAS, v3 software, Germany).

### Organotypic tissue culture

Alginate scaffold disc of approximately 1 mm thickness and 6 mm of diameter were added in groups of 2–4 per well in six-well plates. Tissue slices of approximately 5 ×5 mm were loaded onto the scaffolds and 1 mL of Dulbecco’s modified Eagle’s medium (DMEM, Biowest, France) supplemented with 10% fetal bovine serum (FBS, Lonza, Spain), 100 U/mL penicillin, 100 µg/mL streptomycin and 2 mM L-Glutamine (Sigma-Aldrich) was added to each well. A droplet of medium was added on top of the tissue slice to create a thin film of liquid that helps to maintain the explant humid^20^. Tissue culture was performed at 37 °C in a 5% CO_2_ humidified incubator. Medium was changed every day. Tissue slices from 6 tumours were cultured on scaffolds during 3 weeks to assess for cell morphology, IHC staining and analysis of driver alterations throughout the culture period. For 5 tumours, we obtained 36 tissue slices for sex steroid stimulation at 24 hours (12 slices) 48 hours (12 slices) and 72 hours (12 slices). Three biological replicates were analyzed for each condition: vehicle, estrogen, progesterone and estrogen plus progesterone.

Tissue slices from tumours prior to culture (T0) and from each time point, were submerged in RNAlater and formalin for protein and nucleic acid isolation and for histology and immunohistochemistry (IHC) analysis, respectively.

### Ovarian hormones stimulation

To abrogate the menstrual cycle-dependent influence on the biological characteristics of cells, slices were cultured 2 days in phenol red-free DMEM (Lonza) supplemented with 10% charcoal-treated FBS (Lonza), 100 U/mL penicillin, 100 µg/mL streptomycin and 2 mM L-Glutamine. On the third day, tissue explants were treated with 3.67 × 10^−8^ M of 17β-Estradiol (E2, Sigma-Aldrich), 3.18 × 10^−7^ M of Progesterone (P4, Sigma Aldrich), or combination of both (E2 + P4) and vehicle (unstimulated control) for 24, 48 and 72 hours. The concentrations of sex steroids used were within the physiological tissue concentrations noted in leiomyoma and myometrium^38^. Steroids were initially prepared as stock solutions at 10^−3^ M in absolute ethanol and were stored at −20 °C. Working dilutions were prepared in phenol red-free DMEM medium immediately at the start of the experiment and were stored in ice. All cultures including the controls contained equal amounts of the vehicle, with the final ethanol concentration being 0.002%.

### Nucleic acid and protein isolation

To avoid degradation, tissue sections were immersed in RNAlater and kept at 4 °C until processed. Tissue slice was placed on lysis matrix D (MP Biomedicals, Europe) containing 500 µL RNAtidy G (PanReac AppliChem ITW companies, Darmstadt) and homogenized using FastPrep-24^TM^ 5 G instrument (MP Biomedicals) twice for 30 s with a speed of 6 m/s. The sample tubes were placed on ice for 5 min between pulses. Then, chloroform was added to the lysate, centrifuged and the top aqueous phase was precipitated with isopropanol to isolate RNA according to the manufacturer’s instructions. To the remaining solution, 100% ethanol (3:10 with RNAtidy G) was added, centrifuged 5 min at 2000 × g and the phenol-ethanol phase was moved into a new tube for protein isolation. The pellet containing DNA was washed 2x with 500 µL of sodium citrate 0.1 M/10% ethanol and resuspended in TE. To the protein extract, 2 volumes of isopropanol were added, washed 2x with ethanol 95%, dried and resuspended in lysis buffer (20 mM EDTA, 140 mM NaCl, 5% SDS, 100 mM Tris pH 8.0 and protease inhibitors cocktail, Roche) at 50 °C for 1–3 hours until their complete solubilization^39^.

Residual genomic DNA was removed by incubating the RNA samples with RNase free DNase I and RNasin (Promega Corp. Madison, WI, USA) according to the manufacturer’s instructions. The effectiveness of the DNase treatment was assessed in samples with no reverse transcriptase added (RT-negative). Integrity of RNA was checked by agarose gel electrophoresis. Finally, RNA was quantified by absorbance using a NanoDrop ND-1000 spectrophotometer (ThermoFisher Scientific, Waltham, MA, USA).

### *MED12* mutation detection

Amplification of *MED12* DNA was performed with primers located in intron 1 and intron 2 covering the hot spot region where 99% of mutations have been described (Supplementary Table S2). To check for the presence of mutations in cDNA, a primer pair located in exon 1 and exon 2 was used^40^.

PCR mixes contained 0.25 µM of each primer, 1 unit of Biotaq DNA Polymerase (Bioline, London, UK), 2 mM MgCl2, 150 µM dNTPs and 5 µl of DNA (5–50 ng) or cDNA (1/50 dilution) in a final volume of 20 µl. The cycling conditions were 95 °C for 2 min followed by 40 cycles of 95 °C for 10 seconds, 60 °C for 20 seconds, 72 °C for 20 seconds and a final extension step of 72 °C for 10 minutes.

The PCR products were separated by agarose gel electrophoresis, and the amplicon sizes were verified by comparison with a 100-bp DNA ladder.

PCR products were cleaned up by ExoSAP treatment using Illustra™ ExoProStar™ 1-Step (GE Healthcare Biosciences, Pittsburgh, PA, USA) following the manufacturer’s instructions. Sequencing reactions were performed for both strands with the same primers used in the amplification at the Genomic Service of the University of La Laguna (SEGAI).

For each sample forward and reverse electropherograms were checked manually, edited and assembled using MEGA6^41^. Sequence alignment was performed using CLUSTAL W^42^ as implemented in MEGA6.

### Quantitative polymerase chain reaction (qPCR)

Retro-transcription was carried out using 1 μg of RNA, and first strand complementary DNA was synthesized using Moloney murine leukemia virus reverse transcriptase, RNase H Minus, Point Mutant according to manufacturer’s instructions (Promega Corp.). A Light-Cycler 480 Real-Time PCR detection system apparatus was used to perform the quantification of all transcripts. Each sample was analyzed in triplicate in a total reaction volume of 5 µL consisting of a 2.25 µL 12-fold dilution of cDNA, 1x qPCRBIO SyGreen Mix Lo-Rox (PCRBiosystems, London, UK) and 0.25 µM of each primer. The cycling conditions were 95 °C for 2 min followed by 40–45 cycles of 95 °C for 10 s, 60 °C for 20 s and 72 °C for 30 s. For each experiment, a non-template reaction was included as a negative control. The specificity of the PCR reactions was confirmed by melting curves analysis of the products as well as by size verification of the amplicon in a conventional agarose gel.

Plates data were imported into the GenEx ver 6.1.1.550 data analysis software (MultiD Analyses AB) and interpolate calibration with 3 samples were used to normalize the plates to eliminate run-to-run variation. Data were represented as individual triplicate runs and as averages triplicates (with outliers excluded). Grubbs test was used to remove outliers from each group. The relative expression of each gene was calculated using the 2^−ΔΔCt^ method normalized to expression levels of two reference genes (*GNB2L* and *PUM1*) stably expressed^43^ and to reference (vehicle) control. Log2 transformation of fold-change was used for statistical analysis. Primers sequences are provided in Supplementary Table S3.

### Western blots

Protein concentration was determined using Micro BCA^TM^ protein assay kit (ThermoFisher Scientific). 20 µg of total protein extracts were loaded on 12% sodium dodecyl sulfate-polyacrilamide gel electrophoresis (SDS-PAGE) and then transferred onto polyvinyldifluoride membranes (Bio-Rad, Spain) for 90 min at 350 mA constant. Membranes were blocked with 5% non-fat dry milk in TBS (100 mM Tris, 0,9% NaCl, pH 7,5) containing 0,1% Tween 20 (TBS-T) during 1 h. The membrane was incubated with one of the following mouse monoclonal anti-human antibodies: anti-BCL2 (Santa Cruz Biothecnology, sc-7382; 1:300), anti-PCNA (Santa Cruz Biotechnology, sc-56; 1:100), anti-EGF (Santa Cruz Biotechnology, sc-1667799; 1:200), anti-PGR (Santa Cruz Biotechnology, sc-166169; 1:200), and anti-β-actin (Santa Cruz Biotechnology, sc-47778; 1:1200), overnight in a wet chamber at 4 °C. After three washes in TBS-T, membranes were incubated with mouse-IgGk-BP-HRP secondary antibody diluted 1:2000 (sc-516102, Santa Cruz Biotechnology) for 1 h at 4 °C. After washing, protein bands were developed using an ECL chemiluminescence detection kit (Western bright ECL, Advansta Inc., Menlo Park, CA, USA) and automated image capture of blots was achieved using ChemiDoc XRS + system (Bio-Rad).

Densitometric analysis of protein bands was performed by using Image J program, version 1.52a software (NIH). Integrated optical density for each band was calculated, and then each band was normalized with the integrated optical density of the corresponding β-actin band. Fold difference between relative density units of treated and untreated samples were then calculated.

### Histology and Immunohistochemistry evaluation

Leiomyoma and matched myometrium samples were fixed in 10% buffered formalin, then embedded in paraffin and cut in 3-μm-thick sections. The sections were deparaffinated, hydrated and hematoxylin-eosin (H&E) stained to assess the morphological integrity of tissue samples.

For IHC, deparaffinized 3-μm sections were rehydrated in 0.05 M Tris-buffered saline (TBS), which was used for all further incubations and washes. The sections were incubated overnight at room temperature in TBS buffer containing 0.2% Triton X-100 with one of the following mouse monoclonal anti-human antibodies: anti-estrogenic receptor-α (Santa Cruz Biotechnology, CA, USA, sc-8002; 1:50), anti-progesterone receptor (Santa Cruz Biotechnology, sc-166169; 1:50), anti-desmin (Dako, Carpinteria, CA, IR606). After rinsing, the sections were incubated with biotinylated goat anti-mouse antibody (1:1000; Jackson ImmunoResearch, PA, USA) followed by a streptavidin–peroxidase conjugate (1:1000; Jackson ImmunoResearch), both for 60 min at room temperature. Peroxidase activity was detected using 0.02% 3,3′-diaminobenzidine tetrahydrochloride (Sigma-Aldrich) in Tris-HCl buffer (pH 7.6, 0.05 M) containing 0.01% hydrogen peroxide at room temperature. No primary antibody added was used as control to determine the specificity of the immunostaining. Sections were examined under a light microscope (Leica DM4000B, Leica Microsystems, Germany) and images acquired using a digital camera (Leica DFC300FX).

### Quantitative analysis of the immunoreactive cells

ESR1, PGR and DES immunoreactive cells per microscopic field were counted using computer-based image analysis software (ImageJ, NIH, Bethesda, MD). Five randomly selected fields were digitized on each slide, and then the immunoreactive cells were counted at 400 magnifications. Depending on the immunoreaction, cell profiles with immunostained cytoplasm and unstained nucleus or cell profiles with immunostained nucleus and unstained cytoplasm were counted in each case. Further, stained fragments and artifacts were excluded by the program itself, applying a threshold for minimum object size. Intensity of immunolabeling, evaluated in arbitrary units of gray levels ranging from 1 (black) to 256 (white), was used as criterion for cell immunoreactivity assessment. Cells were considered reactive with values above the ones of their background control sections.

### Statistical analysis

GraphPad Prism v. 5.0 (GraphPad Software, La Jolla, CA, USA) was used for all statistical analysis. A one-way repeated measures ANOVA was used for cell counting across time, followed by Dunnett’s multiple comparison test to compare means for different times to that of the baseline group (T0). qPCR and western blot data were analysed using paired t-test (one-tailed P-value) after assessing that datasets passed normality tests. A one-tailed Wilcoxon matched-pairs signed-ranks test was used when dataset did not pass normality test. For all analysis, P < 0.05 was considered to be statistically significant.

## Supplementary information

Supplemetary Information.

## Data Availability

The data that support the findings of this study are available from the corresponding authors on reasonable request.
