# Supplementary material for: Organotypic culture as a research and preclinical model to study uterine leiomyomas
Source: Sci Rep. 2020 Mar 23;10:5212. doi: 10.1038/s41598-020-62158-w (PMC7090073; doi:10.1038/s41598-020-62158-w)

**Organotypic culture as a research and preclinical model to study uterine leiomyomas**.

Ana Salas, Judith López, Ricardo Reyes, Carmen Évora, Francisco Montes de Oca, Delia Báez, Araceli Delgado* and Teresa A. Almeida.*

Departamento de Bioquímica, Microbiología, Biología Celular y Genética, Universidad de La Laguna. Facultad de Ciencias. Sección de Biología. Avda. Astrofísico Fco. Sánchez s/n. 38200. San Cristóbal de La Laguna, Tenerife, Spain

**Supplementary Figure S1:** Cartoon detailing different steps involved in organotypic culture procedure.

**
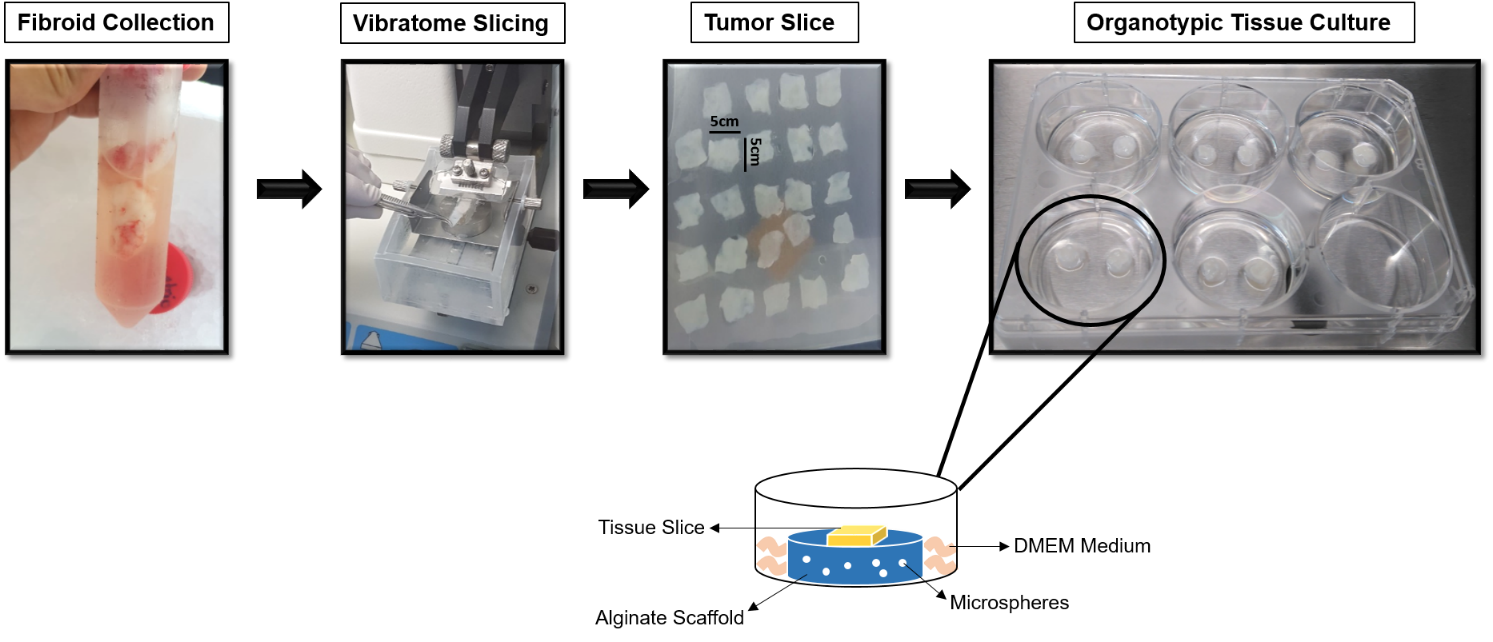
**

**Supplementary Figure S2**: Analysis of positive cells for DESMIN, PGR and ESR1 in five randomly selected fields at baseline (T0) and after 2 (T2), 7 (T7) and 10 (T10) days of culture. *p < 0.05, **p < 0.01. **
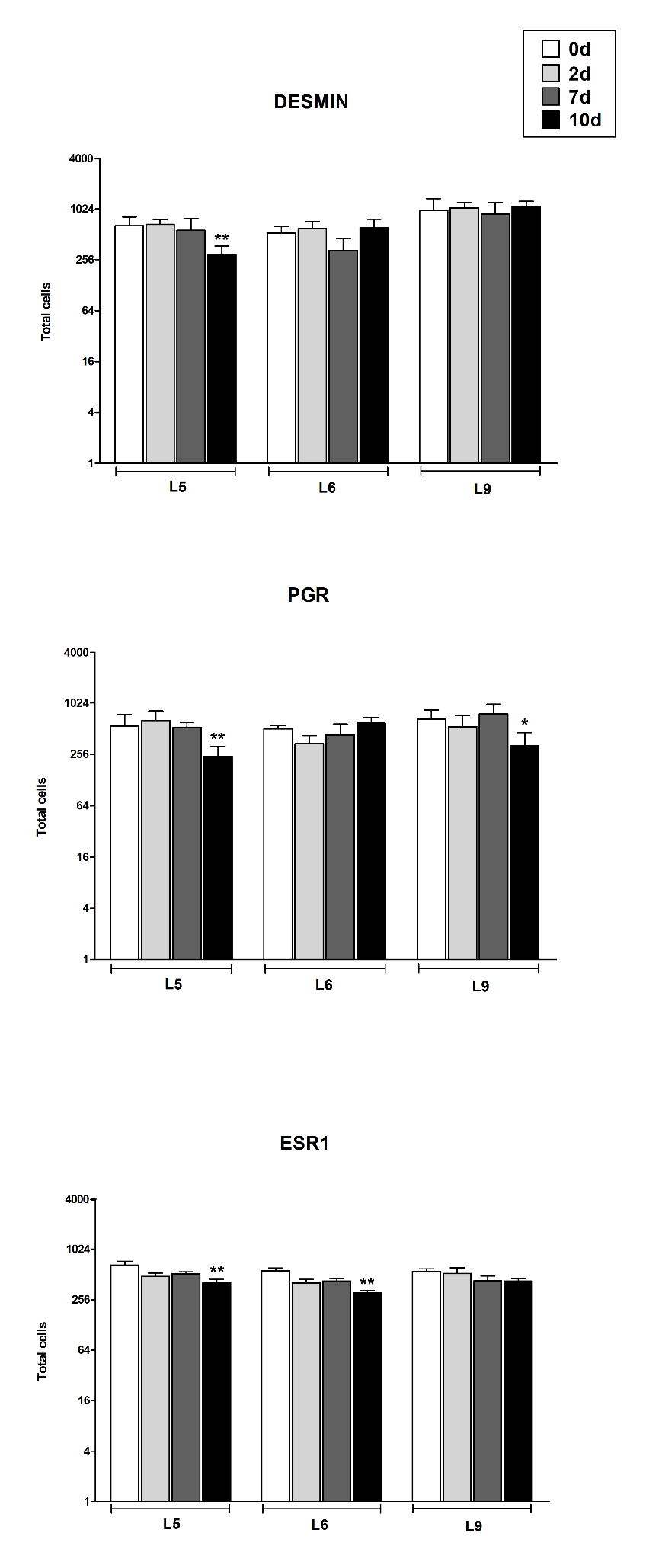
**

**Supplementary Table S1**: Clinicopathological features of 9 smooth muscle tumors evaluated for *MED12* exon 2 mutations and *HMGA2* expression compared to matched myometrium.

| **Tumor ID** | **Age** | **Size (cm)** | **Location** | **MED12 status** | **HMGA2 Expression** | **Experimental Procedure** |
| --- | --- | --- | --- | --- | --- | --- |
| L5 | 39 | 5 | Subserous | c.131 G>A (p.G44D) | Up | Long term culture |
| L6 | 39 | 4 | Submucous | c.128 A>C (p.Q43P) | No | Long term culture |
| L8 | 41 | 4 | Intramural | c.142_157del16insA (p.Gln48_Gly53delinsArg) | No | Long term culture |
| L9 | 49 | 7 | Intramural | c.130 G>C (p.G44R) | No | Long term culture |
| L10 | 44 | 5 | Intramural | WT | Up | Long term culture |
| L11 | 40 | 6 | Intramural | WT | No | Long term culture |
| L20 | 47 | 5 | Subserous | c.131 G>A (p.G44D) | No | Hormonal Treatment |
| L21 | 47 | 6 | Intramural | c.130 G>A ( p.G44S) | No | Hormonal Treatment |
| L23 | 58 | 12 | Intramural | WT | Up | Hormonal Treatment |
| L24 | 47 | 7 | Intramural | c.130 G>A ( p.G44S) | No | Hormonal Treatment |
| L25 | 48 | 4 | Subserous | c.131 G>A (p.G44D) | No | Hormonal Treatment |

**Supplementary Table S2.** Sequence of primer pairs used for qPCR.

| Gene Name | Forward (5’-3’) | Reverse (5’-3’) | Size (pb) |
| --- | --- | --- | --- |
| *BCL2* | TCCAGGATAACGGAGGCTG | GGCCAAACTGAGCAGAGTC | 107 |
| *CCND1* | TTGTGTGTATCGAGAGGCCA | CCAGAAATGCACAGACCCAG | 118 |
| *COL1A1* | GAGGGCCAAGACGAAGACATC | CAGATCACGTCATCGCACAAC | 140 |
| *ESR1* | GAAAGGTGGGATACGAAAAGACC | GCTGTTCTTCTTAGAGCGTTTGA | 163 |
| *HMGA2* | AGAGTCCCTCTAAAGCAGCTCA | CAACTGCTGCTGAGGTAGAAATCG | 196 |
| *IGF1* | GCTCTTCAGTTCGTGTGTGGA | GCCTCCTTAGATCACAGCTCC | 133 |
| *IGF1R* | CGTGAAGATCCGCCATTCTC | TCACTTCCTCCATGCGGTAA | 236 |
| *PGR* | AAGGAGTTGTGTCGAGCTCA | GTTTCACCATCCCTGCCAAT | 198 |
| *VEGFA* | CAAAAACACAGACTCGCGTTG | TGAGAGATCTGGTTCCCGAA | 134 |
| *GNB2LI* | GAGTGTGGCCTTCTCCTCTG | GCTTGCAGTTAGCCAGGTTC | 224 |
| *PUM1* | GACGCTATGGTGGACTACTTCT | TGGAACGCACCTGATGTTCTG | 142 |
| *MED12-DNA* | GCCCTTTCACCTTGTTCCTT | TGTCCCTATAAGTCTTCCCAACC | 291 |

**Supplementary Figure S3**: Full length blots of the five tumours with the 3 biological replicates for the 4 conditions, vehicle (C), estrogen (E), progesterone (P) and estrogen plus progesterone (EP) after 24 h, 48 h and 72 h of stimulation. After transfer, the blot was cut in three pieces according to the pre-stained ladder. Top piece was incubated sequentially with 2 different antibodies, EGFR and PGR (after stripping and re-probing), middle piece was incubated sequentially with 2 different antibodies, ACTIN and PCNA (after stripping and re-probing) and bottom piece was incubated with BCL2. Images shows a merge picture of the chemiluminescent blot image with a colorimetric image of the same blot piece to determine ladder bands. Expected molecular weights are: ACTIN 42 kDa, BCL2 26 kDa, EGF 160 kDa, PCNA 35 kDa, PGR isoform A 81 kDa, PGR isoform B 116 kDa. Red lines on blot indicate size of the ladder band.


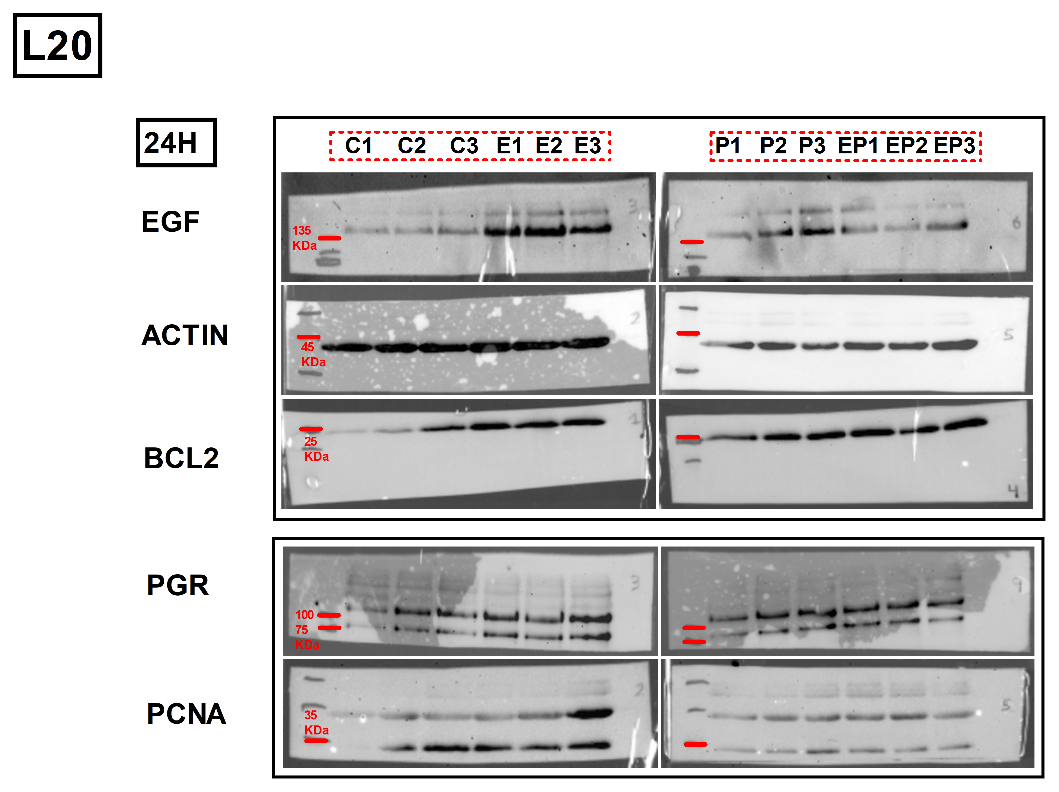


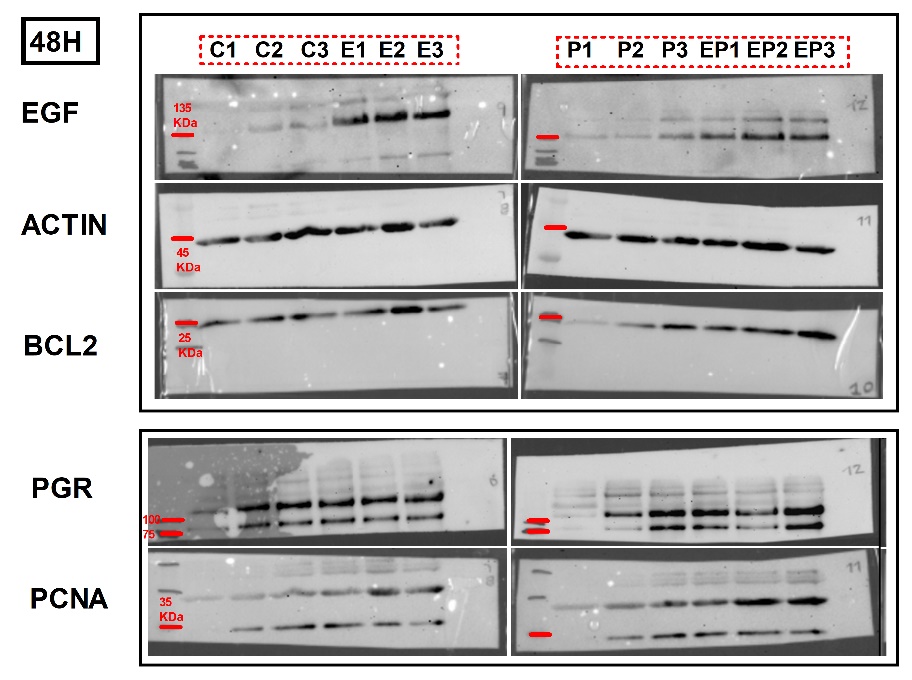


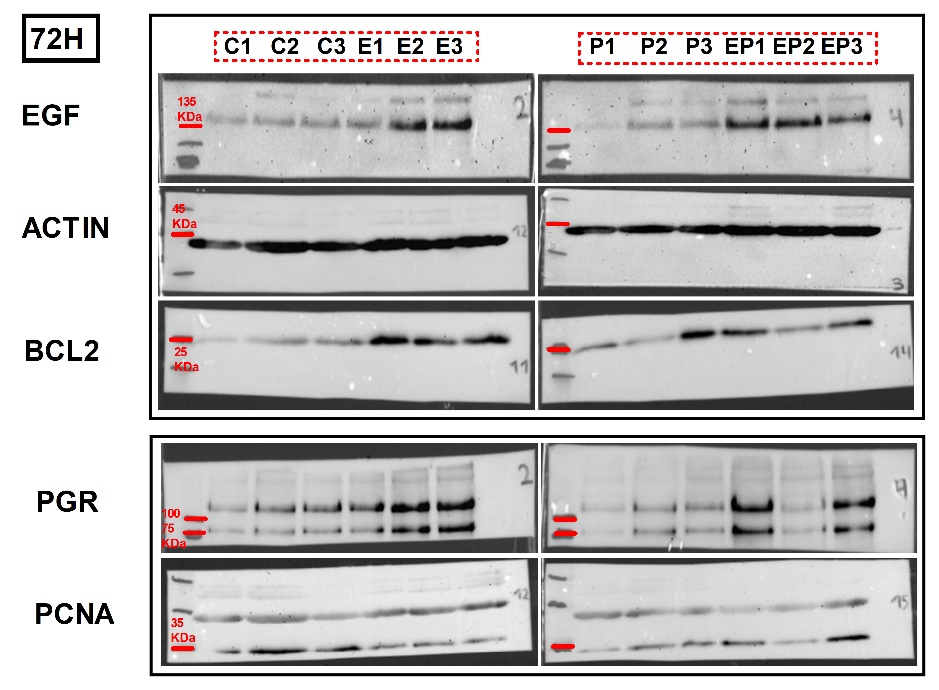


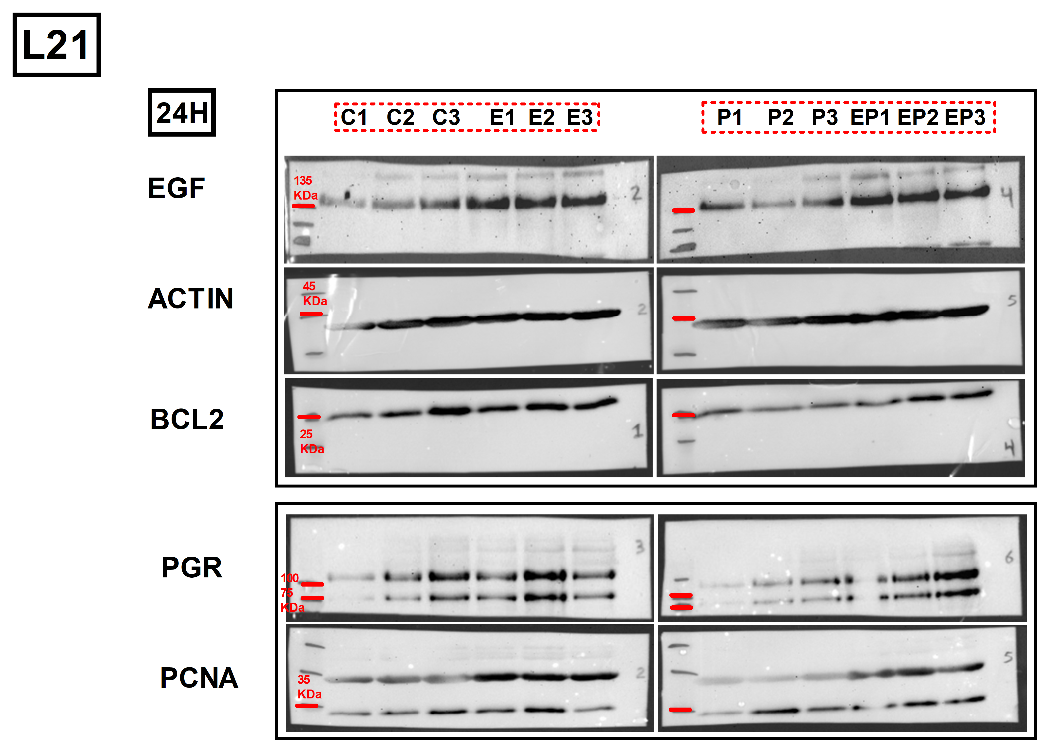


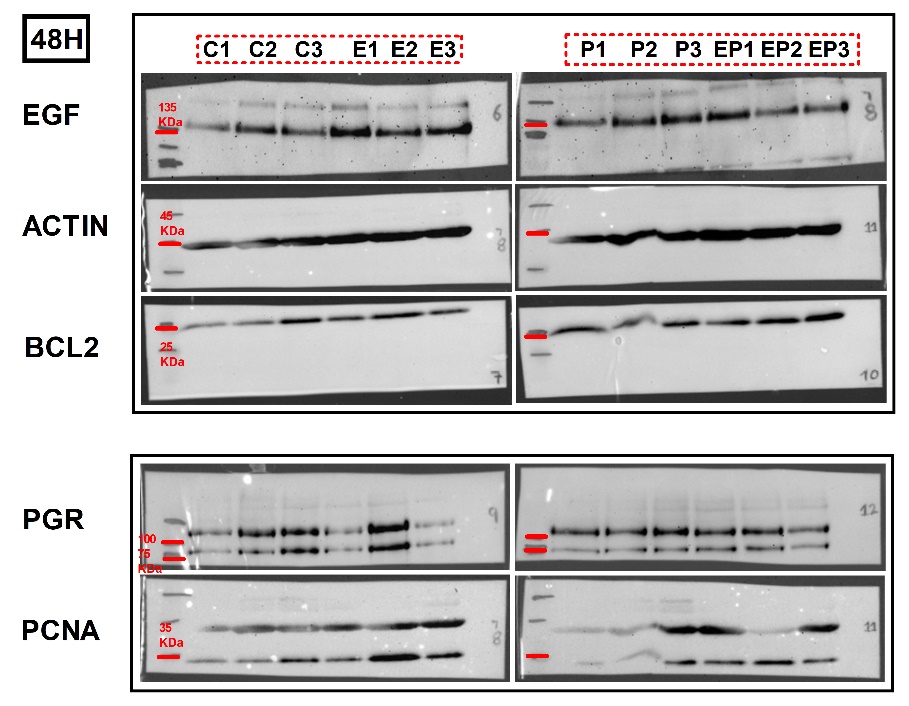


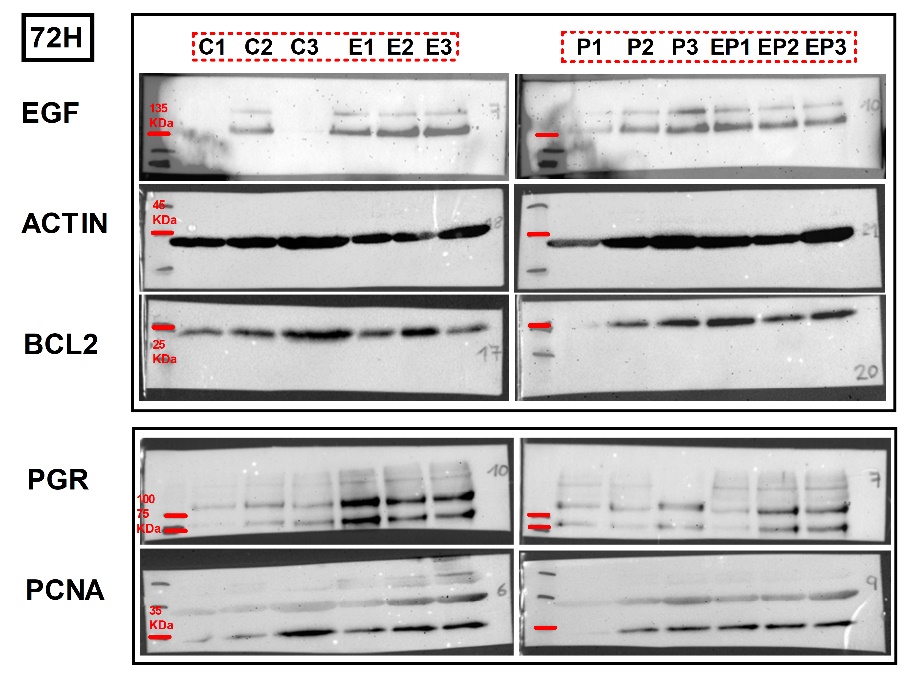


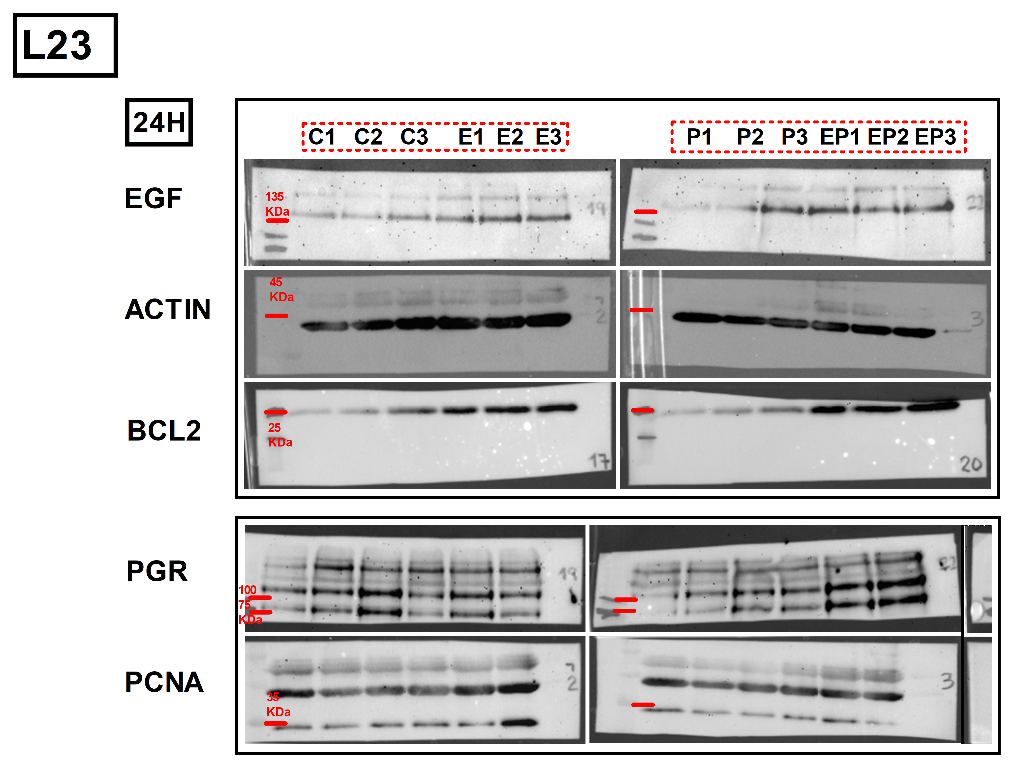


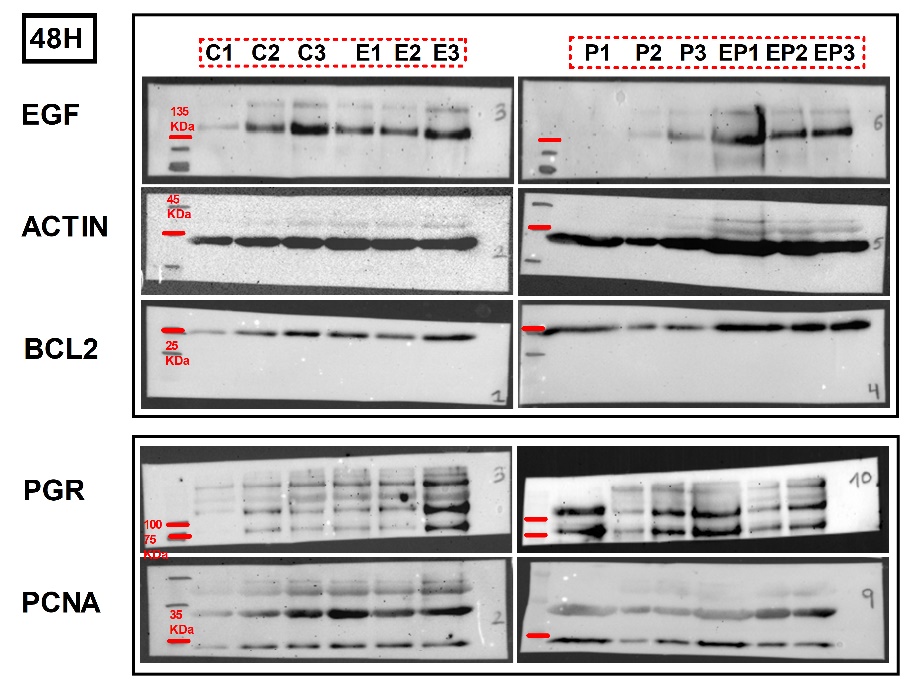


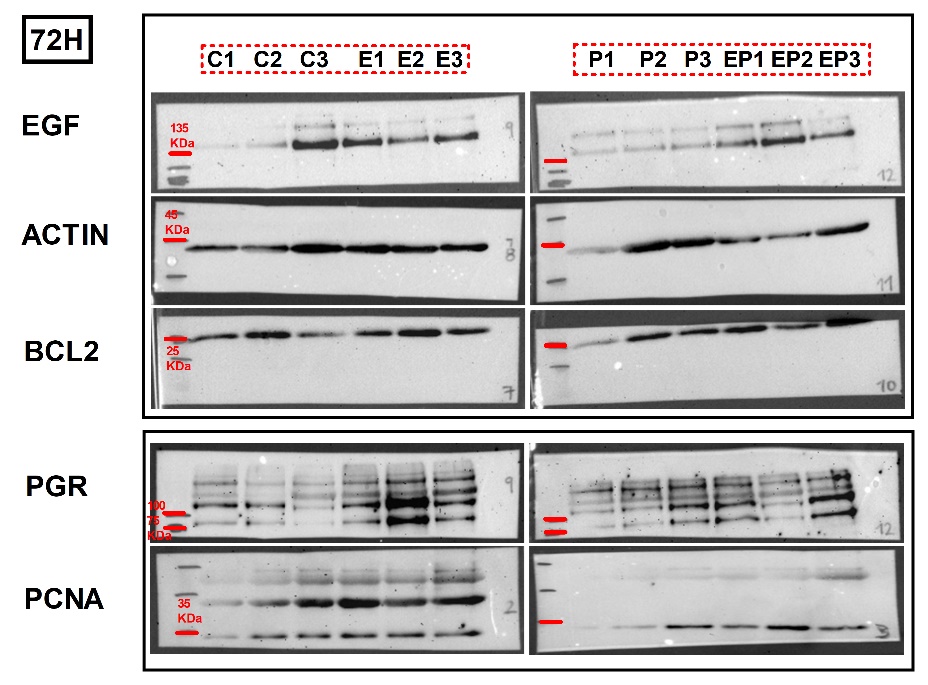


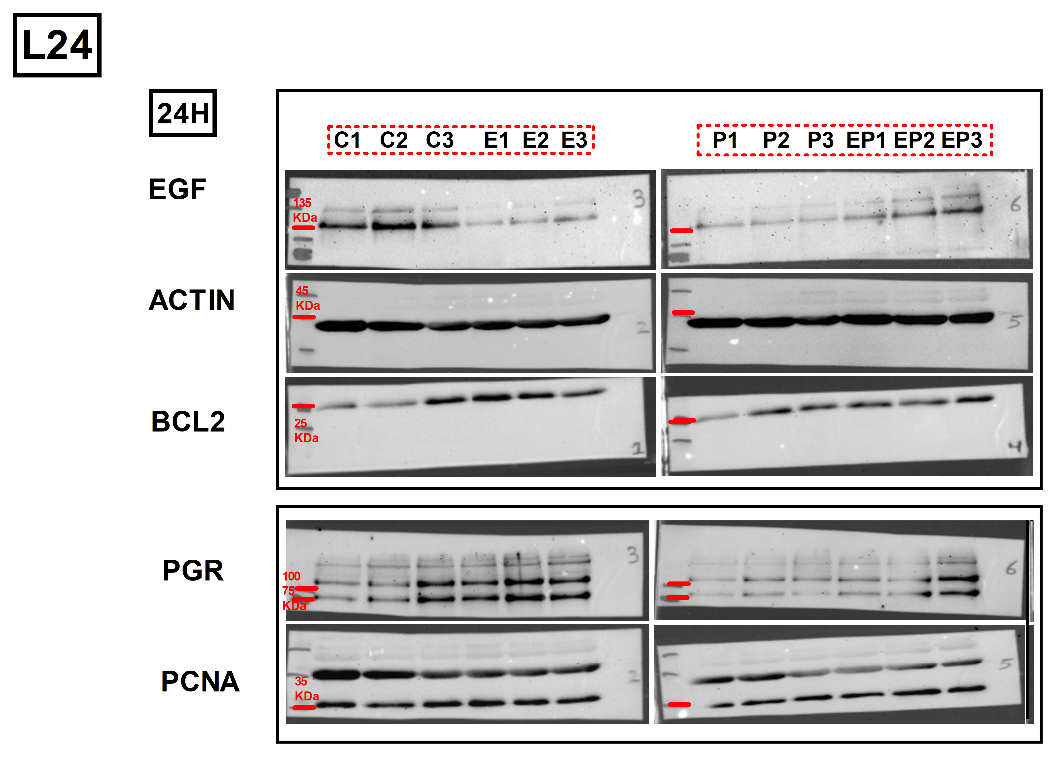


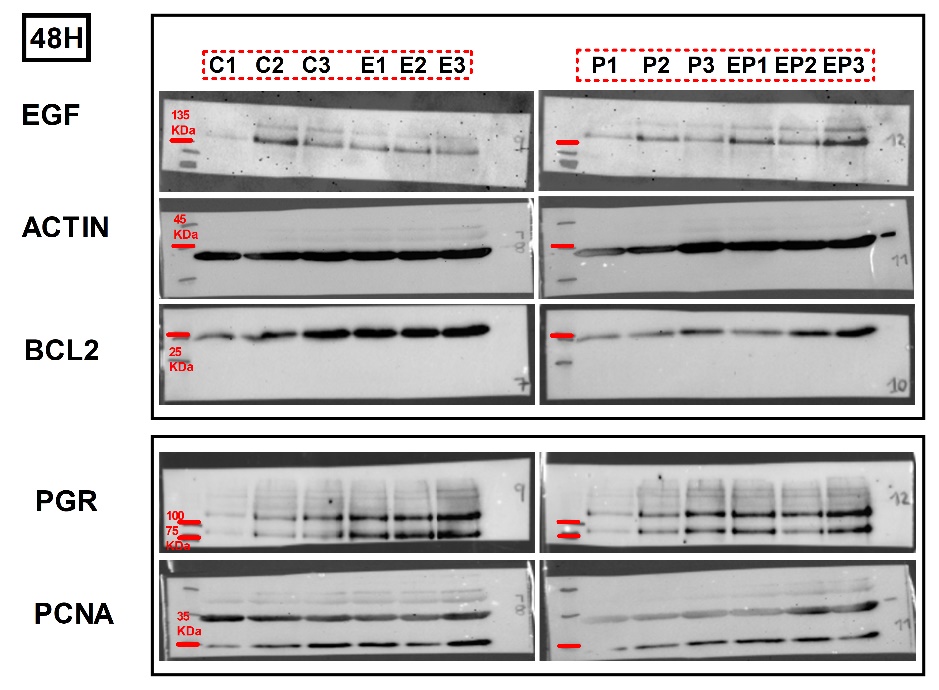


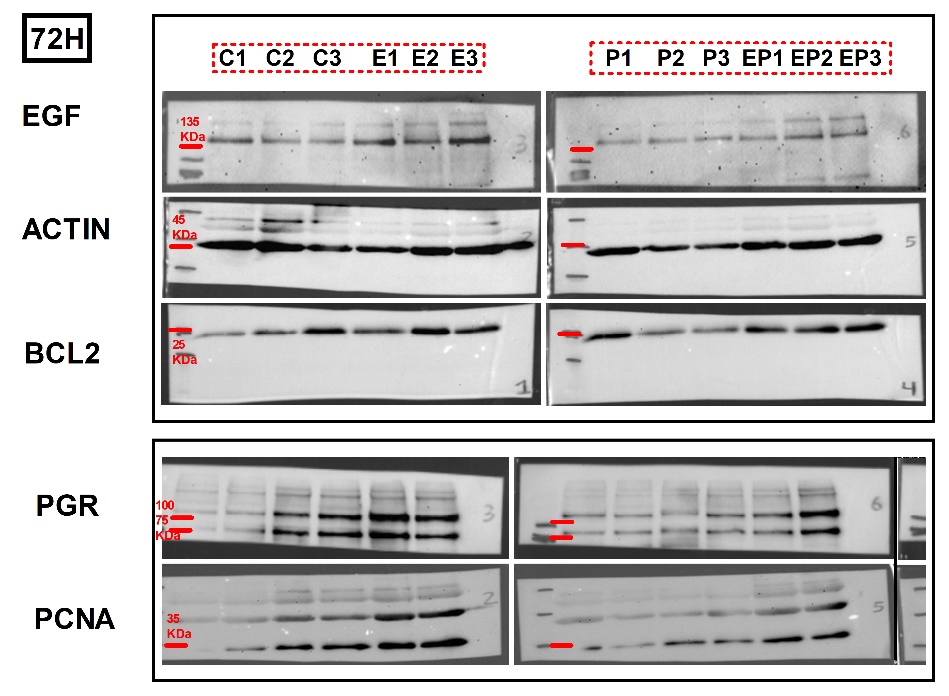


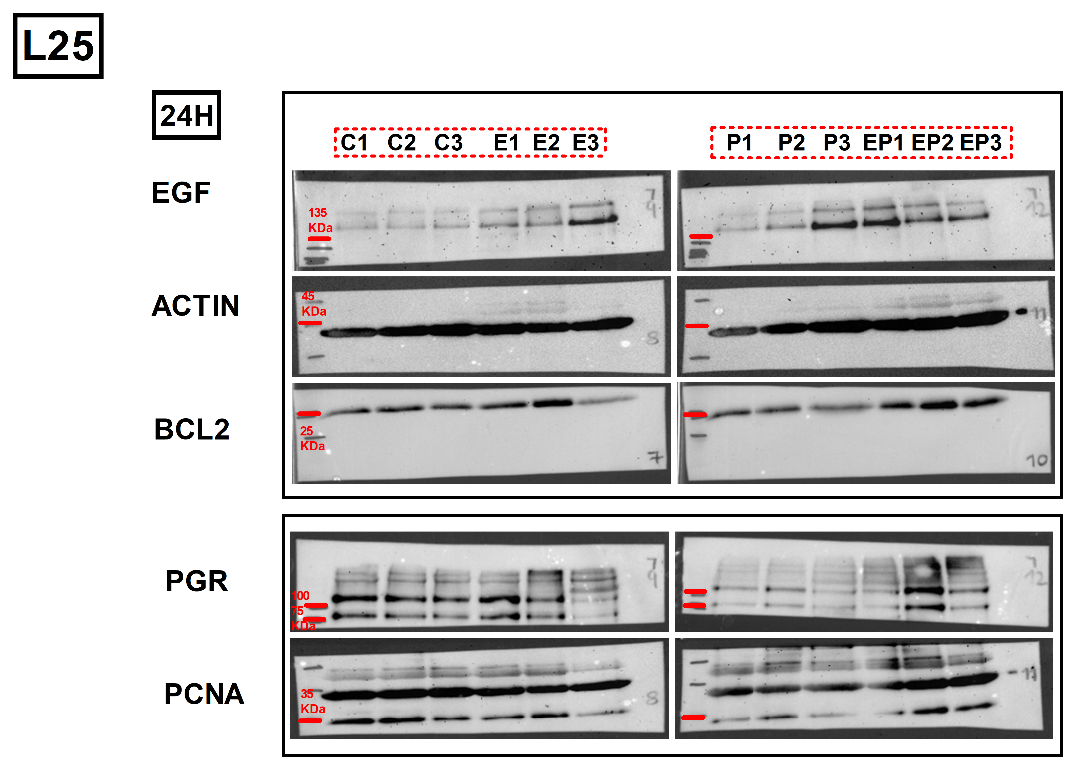


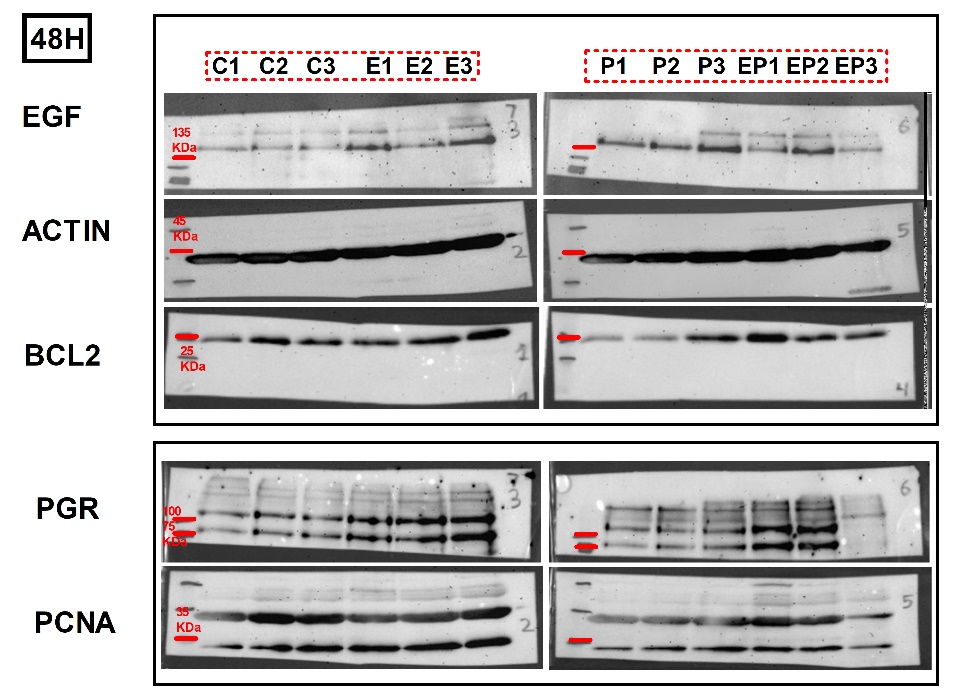


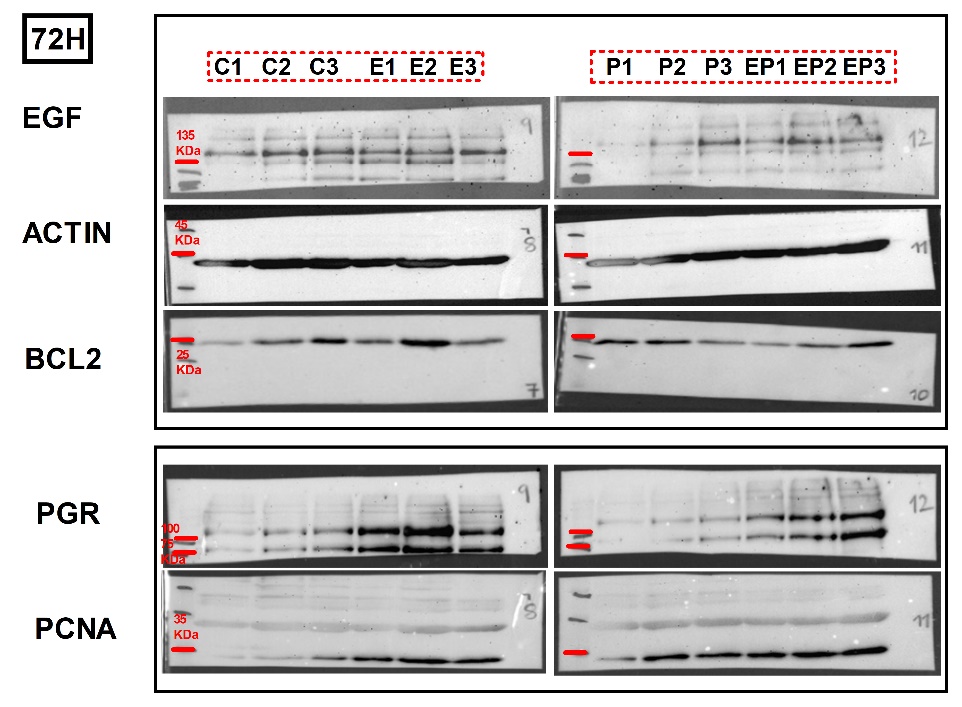

Supplement: Supplementary file 1 — Supplemetary Information. [file 41598_2020_62158_MOESM1_ESM.docx]
